# Effects of Microcurrent on Oxygen Saturation by Controlling Rectus Abdominis Activity in Preterm Infant With Desaturation During Feeding: A Pilot Study

**DOI:** 10.3389/fped.2021.694432

**Published:** 2021-11-22

**Authors:** Dong Rak Kwon, Dae Gil Kwon, Ji Eun Jeong

**Affiliations:** ^1^Department of Rehabilitation Medicine, Catholic University of Daegu School of Medicine, Daegu, South Korea; ^2^Department of Pediatrics, Catholic University of Daegu School of Medicine, Daegu, South Korea

**Keywords:** desaturation, feeding, preterm, infant, rectus abdominis, microcurrent

## Abstract

**Objective:** To determine whether a portable microcurrent therapy device (PMTD) of the rectus abdominis muscles is effective for treating desaturation during feeding in preterm infants and to evaluate the association between initial electrical activity of respiratory muscle and long-term development delay.

**Methods:** Twenty preterm infants with desaturation during feeding were recruited. Respiratory muscle activity was quantified by calculating the root mean square (RMS) of the electromyography. All preterm infants received a 30 min PMTD application to the rectus abdominis and diaphragm daily for 2 weeks. RMS of diaphragm and rectus abdominis, feeding volume, frequency of desaturation during feeding at baseline (pre-PMTD) and 1, 2 week post-PMTD were measured. The number of days it took to treat desaturation after PMTD was measured. A Denver developmental screening test was performed and infants were divided into 3 groups: (1) normal; (2) caution; and (3) delayed at 3months after PMTD.

**Results:** The desaturation during feeding of all the preterm infants subsided after PMTD and the mean days took to treat desaturation was 25.4 ± 14.2 days. The RMS of diaphragm, rectus abdominis, and frequency of desaturation during feeding were significantly decreased and the feeding volume was significantly increased after PMTD (*p* < 0.01). The mean treatment duration for desaturation was negatively correlated with RMS of rectus abdominis at baseline and 1 week post-PMTD, respectively (Pearson's correlation coefficient = −0.461,−0.514, *p*-value = 0.047, 0.029). RMS of rectus abdominis of Group 3 is lower than that of group 1 and 2 (*p* < 0.01).

**Conclusions:** This pilot study showed that the microcurrent therapy of rectus abdominis is an efficient therapy for the treatment of preterm infants with desaturation during feeding, especially preterm infants with higher activity of the rectus abdominis. In preterm infants with lower rectus abdominis activity, longer time is required to treat desaturation by microcurrent therapy and developmental delay is observed at months post-treatment.

## Introduction

In premature infants, coordination of sucking, swallowing, and breathing rhythms is critical for successful suckle feeding (1, 2). Immature coordination, gastroesophageal reflux, immature oral structures, and a combination of conditions can increase the risks of desaturation while feeding (3–9). A clinically significant oxygen desaturation phenomenon occurs when there is any reduction in oxygen saturation < 90% for 1 second or longer (10). Moreover, although preterm infants can also experience impaired lung function during gavage feeding, desaturation can occur more frequently during bottle feeding, particularly when there is an extant gavage tube (3, 11–14). Premature infants are frequently fed using these methods. Immature incoordination of suck-swallow-breathing can cause frequent desaturation, which can affect multiple organs (e.g., heart, lungs, and brain), impacting the subsequent infant growth and development (15–18).

Evidence suggests that diaphragmatic fatigue is involved in the pathogenesis of desaturation in premature infants (19–21). Paradoxical breathing is common in infants and is particularly notable in preterm infants as a result of their highly compliant chest wall (4). Reports suggest that this defect enhances volume displacement of the diaphragm during inspiration and requires substantial effort and energy, as well as promoting diaphragmatic fatigue and desaturation. Gewolb and Vice (22) demonstrated that hypoxemic episode duration and severity were related to simultaneous abdominal muscle contractions following mechanical ventilation in preterm infants. Moreover, delayed lung inflation following abdominal muscle contraction eventually results in a reduction in the lung volume below baseline.

Furthermore, a previous study reported that preterm infants with desaturation during feeding showed high electrical activity in the diaphragm and rectus abdominis muscle (23). They assumed that excessive contraction of diaphragm and rectus abdominis muscle lead to diaphragm fatigue and feeding desaturation. Therefore, they suggest that reducing the excessive activity of abdominal muscle may effectively control feeding desaturation in preterm infants.

Microcurrent electrical stimulation represents a physical modality for the delivery of current in the microampere range. No adverse effects have been associated with microcurrent stimulation as it works at the microampere level and simulates the electrical intensity of living tissue (24–28). A previous study demonstrated that microcurrent therapy markedly reduced the muscle electrical activity and increased muscle power efficiently through minimal muscle recruitment (29).

Taking into account the importance of safe and effective treatment for preterm infant with desaturation during feeding, and in light of the aforementioned issues: we aimed to explore the association between initial electrical activity of respiratory muscle and long-term development delay and the efficacy of microcurrent treatment in these patients. To our knowledge, this preliminary report is the first to investigate this convenient treatment method in preterm infant with desaturation during feeding.

## Materials and Methods

### Participants

This was a prospective, single arm, pilot study. A total of 79 preterm infants were enrolled with desaturation during feeding who underwent treatment in neonatal intensive care unit, 59 of which the desaturation resolved spontaneously before 35 weeks of gestation. There were 20 preterm infants with respiratory distress syndrome referred from the pediatrics department to rehabilitation medicine department regarding desaturation during feeding. These infants were recruited to the present study, between May 2015 and March 2016 (Table 1).

**Table 1:** Demographic data.

**Variable**	**Value**
GA at birth (week)	29.1 ± 4.1
GA at study (week)	37.0 ± 1.3
Weight at birth (g)	1366.5 ± 675.7
Weight at study (g)	2483.0 ± 375.7
Sex (M/F)	11/9
Brain lesion (abnormal/normal)	8/12

*Values are represented as the mean ± standard deviation or numbers. GA, Gestational age*.

Significant feeding desaturation was defined as an arterial oxygen saturation level using a pulse oximeter < 85% for longer than 2 s (moderate to severe desaturation) (10, 16, 23). None of the infants exhibited concurrent sepsis or craniofacial anomalies. All infants were bottle-fed during their scheduled feeding times by a skilled nurse with over 2-3 years of experience in a neonatal intensive care unit. In infants aged < 3 months, a small nipple size (Aissok, Greenmom, Anyang, Korea) was used for each feeding. Gavage-fed infants were excluded from the study. The study was approved by the Institutional Review Board and Ethics Committee of University Medical Centre (IRB number: MDCR-15-011). Written informed consent was granted by the parents or guardians of the infants.

### Outcome Measurements

Surface electrodes were used to record the electromyographic (EMG) activity of the diaphragm and rectus abdominis muscle as described previously (1) using a four-channel electrophysiology unit (Medelec, Company of Oxford, Oxford, UK). Surface electrodes were placed in the eighth intercostal space between the mid-clavicular and mid-axillary line to measure the electrical activity of the diaphragm muscles. Surface electrodes were placed 2 cm laterally to the umbilicus to measure the activity of the rectus abdominis muscles. The electromyographic (EMG) activity of the muscles was recorded for 1 h at resting state after feeding. The level of respiratory muscle activity was quantified by calculating the root mean square (RMS) envelope of the EMG signal. The RMS of the diaphragm and rectus abdominis was measured. The brain imaging study (ultrasound or magnetic resonance imaging) was reviewed. Feeding volume at each feed and frequency of desaturation during feeding was measured. Treatment duration was defined as the time between the initial treatment and achieving no desaturation event during feeding. There was a loss to follow up at 3 months after treatment, for 9 infants. In 11 infants, the Denver developmental screening test II (DDST) was performed (6). The DDST includes four developmental elements: social contact, language, fine motor skills, and gross motor skills. Infants were divided into three groups: group 1 (*n* = 4), normal; group 2 (*n* = 4), caution; and group 3 (*n* = 3), delayed, according to guidelines (30, 31).

### Treatment

**Figure 1:**
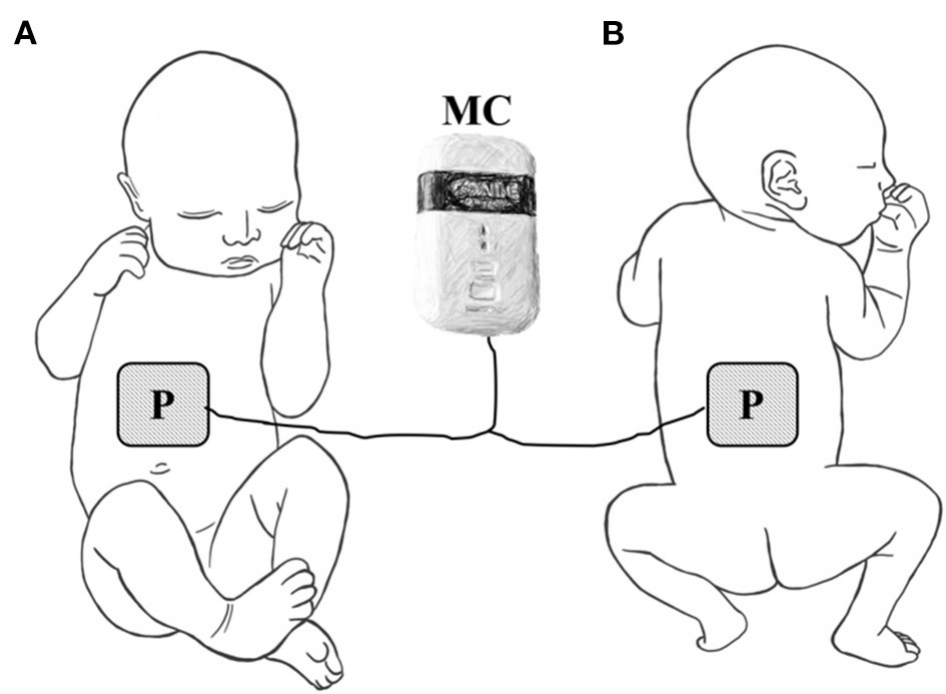
Placement of electrode for microcurrent therapy on **(A)** the front side of the infant or **(B)** the back of the infant. P, electrode patch, MC, microcurrent generator.

All preterm infants received intensive physical therapy and microcurrent therapy in the ICU. Physical therapy consisted of oromotor stimulation, respiratory muscle stretching and relaxation, and trunk stabilization. Microcurrent therapy was applied to the front and back of body to treat diaphragm and rectus abdominus. The front patch placed to 1 cm above the umbilicus on midline. A back patch was placed at the same location on the back (Figure 1). All infants received 2 weeks of daily treatment with a small and portable microcurrent therapy device (intensity, 25μA; frequency, 8 Hz; Granthe, Cosmic Co., Seoul, Korea) for 30 min. The current intensity was significantly lower than each infant's sensation threshold. The microcurrent generator provided an alternating current using a monophasic rectangular pulse format with a reversal in polarity every 2 s.

### Statistical Analysis

A repeated measured one-way ANOVA was used to evaluate the change in EMG activity. A Pearson's correlation analysis and Spearman's correlation analysis were used to calculate the correlation between numeric variables. The association between DDST and other variables was evaluated using a one-way ANOVA and Kruskal-Wallis test.

## Result

A total of 20 preterm infants (mean age: 37.0 ± 1.3 weeks; mean weight: 2483.0 ± 375.7 g; 11 males and 9 females) were enrolled. There were 8 out of 20 infants with brain lesions, including four with periventricular leukomalacia, two with intraventricular hemorrhage, and two with grade I germinal matrix hemorrhage. All preterm infant's desaturation during feeding had subsided after treatment. The mean duration of treatment was 25.4 ± 14.2 days.

The RMS of diaphragm and rectus abdominis muscle decreased continuously during treatment (Table 2) (*p* < 0.01).

**Table 2:** Changes in the electrical activity of respiratory muscles and feeding status.

	**Baseline**	**1 weeks**	**2 weeks**	***p*-value**
RMS-D (μV)	280.1 ± 104.7	256.6 ± 85.6	231.8 ± 74.0	<0.01^*^
RMS-R (μV)	58.9 ± 21.4	50.9 ± 18.9	43.9 ± 18.9	<0.01^*^
F-VOL (ml)	53.8 ± 14.2	75 ± 14.6	89.4 ± 15.3	<0.01^*^
DES-FREQ (n)	3.9 ± 2.2	1.8 ± 0.9	0.8 ± 0.8	<0.01^*^

*RMS-D, root mean square of diaphragm; RMS-R, root mean square of rectus abdominis; F-VOL, feeding volume; DES-FREQ, frequency of desaturation during feeding. Values are represented as the mean ± standard deviation*.

* *: p-value < 0.05 by repeated measure one-way ANOVA*.

The mean duration of treatment was negatively correlated with the RMS of rectus abdominis at baseline and 1 week after treatment (Pearson's correlation coefficient = −0.461,−0.514 *p*-value = 0.047, 0.029) (Table 3). RMS of rectus abdominis of Group 3 was lower than that of group 1 and 2 (*p* < 0.01) (Table 4).

**Table 3:** Correlation analysis among the clinical variables and duration of treatment (*n* = 20).

	**Treatment time**
GA (week)	−0.266
WT (gram)	−0.178
APG 1 min	−0.181
APG 5 min	0.055
RMS-D baseline (μV)	−0.027
RMS-D 1wk (μV)	−0.005
RMS-D 2wk (μV)	0.009
RMS-R baseline (μV)	−0.461^*^
RMS-R 1wk (μV)	−0.514^*^
RMS-R 2wk (μV)	−0.407

*GA, gestational age at birth; WT, weight at birth; APG 1m, APGAR score at 1 min; APG 5m, APGAR score at 5min; RMS-D, root mean square of diaphragm; RMS-R, root mean square of rectus abdominis, 1wk; 1 week after PMTD therapy, 2wk; 2 week after PMTD therapy, Treatment time; The mean days took to treat desaturation. Values are Pearson's correlation coefficient or Spearman's correlation coefficient*.

* *: p-value < 0.05, Pearson's correlation analysis*.

**Table 4:** Clinical variables according to development evaluation.

	**Group 1**	**Group 2**	**Group 3**	***p*-value**
	**(*n* = 4)**	**(*n* = 4)**	**(*n* = 3)**	
GA	29.3 ± 3.1	25.3 ± 1.7	28.4 ± 2.3	0.119
WT	1322.5 ± 477.0	782.5 ± 92.2	1063.3 ± 151.8	0.101
APG 1min	4.3 ± 3.4	4.0 ± 1.4	4.0 ± 2.0	0.926
APG 5min	7.8 ± 1.5	6.8 ± 1.3	7.0 ± 1.0	0.764
RMS-D baseline	216.4 ± 84.4	249.8 ± 147.9	287.2 ± 52.8	0.700
RMS-D 1wk	194.0 ± 71.1	219.0 ± 105.2	274.7 ± 28.4	0.440
RMS-D 2wk	170.0 ± 80.6	206.3 ± 89.8	234.2 ± 10.1	0.584
RMS-R baseline	64.8 ± 11.4	82.5 ± 13.6	35.6 ± 11.1	0.003^*^
RMS-R 1wk	58.9 ± 12.5	74.3 ± 9.0	31.6 ± 9.3	0.002^*^
RMS-R 2wk	46.0 ± 6.6	67.5 ± 14.0	24.0 ± 6.1	0.003^*^
Brain lesion (normal/abnormal)	3/1	2/2	0/3	0.139

*GA, gestational age at birth; WT, weight at birth; APG 1 m, APGAR score at 1 min; APG 5m, APGAR score at 5min; RMS-D, root mean square of diaphragm; RMS-R, root mean square of rectus abdominis, 1wk; 1 week after PMTD therapy, 2wk; 2 weeks after PMTD therapy, group 1; normal, group 2; caution, group 3; delayed. p-value: by one-way ANOVA or Kruskal-Wallis test or Chi-square test*.

* *: RMS of delayed group was significantly lower than that of caution or delayed group, by post hoc Tukey test*.

There was no correlation between gestational age, weight, brain lesion, APGAR score, RMS of the diaphragm and treatment duration (Table 3). There was no association between gestational age, weight, brain lesion, APGAR score, RMS of diaphragm and developmental delay (Table 4).

## Discussion

The current study explored the efficacy of microcurrent stimulation of preterm infants with desaturation during feeding. Our preliminary results show that patients had decreased RMS of diaphragm and rectus abdominis muscle and improved oxygen saturation. To the best of our knowledge, these data are the first to report the effectiveness of microcurrent in preterm infants with desaturation during feeding.

Two mechanisms of microcurrent therapy have been suggested regarding the treatment of desaturation during feeding: 1) the therapeutic effect may be related to the maintenance of intracellular Ca2+ homeostasis in muscle (24, 32). Moreover, abdominal muscle contractions in preterm infants may result in forced exhalation, which reduces the lung volume and places a substantial load on the inspiratory muscles. These muscles must then overcome the increase in workload before they can perform effective inspiration using the diaphragm. Additionally, in preterm infants, the diaphragm contains < 10% type I muscle fibers and a low amount of type IIb muscle fibers (33, 34). Together, the lack of fatigue-resistant type I muscle fibers, high proportion of fatigue-susceptible type IIc muscle fibers, and low oxidative capacity of the neonatal diaphragm indicate that the diaphragm muscle may be prone to fatigue (19, 20, 31).

Thus, enhanced diaphragmatic breathing may represent substantial energy expenditure that contributes to ventilatory failure and diaphragmatic fatigue. As a consequence, an imbalance between an imposed load and inspiratory muscle capacity in preterm infants leads to desaturation symptoms. Increased RMS indicates that work-of-breath and thoracoabdominal asynchrony are elevated in preterm infants. It has previously been shown (29) that microcurrent therapy lowers the RMS of hand grip muscle and increases of hand grip strength in the elderly. Thus, the authors suggest that an elevated concentration of intracellular calcium might alter membrane integrity, causing functionally prolonged muscle contraction.

Second, microcurrent therapy may enhance adenosine triphosphate (ATP) synthesis of amino acid transportation, and protein synthesis, which can prevent the vicious cycle of diaphragm and rectus abdominis muscle contraction (35, 36). Prolonged actin-myosin coupling can promote muscle fiber contraction and increase the flow resistance in the microvasculature of the contracted muscle. Arteriolar and capillary constriction impairs blood flow to the tense muscle fibers and can be aggravated by local vasoconstrictor reflex. This can lead to reduced levels of oxygen and glucose, decreasing the regeneration of ATP (37–41). Decreased ATP can interfere with Ca2+ reuptake into the sarcoplasmic reticulum, a process that is partially ATP-dependent, which prolongs actin-myosin cross-bridging and initiates a vicious cycle (42). Previous studies have described the benefits of electric current on soft tissue repair as a result of supplying ATP (43).

The findings of the present study demonstrate that infants with a lower RMS of rectus abdominis take longer time to treat desaturation and developmental delay at 3 months after treatment. These findings completely correlate with the findings of previous studies and we speculate that this consistency may be attributed to the muscle hypotonia. Previous studies (44–47) have demonstrated that hypotonia has a negative impact in infants exhibiting genetic syndromes, reporting difficulties in latching and sucking during infancy and challenges in managing solid foods during childhood (45, 46). Hypotonia can interfere with the development of the motor skills required for the success of oral feeding (47), representing a challenge to the transition through the various developmental stages of feeding.

The infants with a low RMS of the rectus abdominis showed delayed development. Two mechanisms are possible for this observation. First, the infants with the low RMS of the rectus abdominis recovered slowly, therefore, they were exposed to a hypoxic environment for a longer period of time. In addition, chronic hypoxia interferes with brain development and causes developmental delay. Second, rectus abdominis is a trunk muscle essential for gross movement. Low activity of the rectus abdominis can represent an independent factor to affect development.

There are some limitations associated with this study. First, the sample size is too small. Second, our study lacks a control group for the direct comparison of microcurrent therapy to other standard dysphagia rehabilitation therapy since all parents of preterm infants want to receive microcurrent therapy. Third, we did not perform other test to evaluate the cause of desaturation (e.g., chest CT or esophageal pH monitoring). Therefore, we cannot exclude other factors influence desaturation. Therefore, future randomized controlled studies are warranted. Fourth, the duration of follow up is too short to detect developmental delay. Finally, to achieve optimal results, the effects of microcurrents of various frequencies and durations (e.g., <30 min or >3 h.) must be assessed.

## Conclusion

This pilot study shows that microcurrent therapy of the rectus abdominis is an efficient treatment for preterm infants with desaturation during feeding, especially preterm infants with higher rectus abdominis activity. In preterm infants with lower rectus abdominis activity, infants take longer to respond to microcurrent therapy to treat desaturation and they show developmental delay at 3 months after treatment. We can assess the electrical activity of the rectus abdominis muscle in a clinical setting, and it can help determine the cause of desaturation and establish a therapeutic plan, as well as predict the prognosis of development.

## Data Availability Statement

The original contributions presented in the study are included in the article/supplementary material, further inquiries can be directed to the corresponding author/s.

## Ethics Statement

The studies involving human participants were reviewed and approved by the Institutional Review Board of Daegu Catholic University Medical Center (DCUMC) approved this study. Written informed consent to participate in this study was provided by the participants' legal guardian/next of kin.

## Author Contributions

DRK contributed to the study conception and design. DRK, DGK, and JEJ contributed to the interpretation of the study results, writing, and editing of this manuscript. All authors contributed to the article and approved the submitted version.

## Funding

This research was supported by the Basic Science Research Program through the National Research Foundation of Korea (NRF), which is funded by the Ministry of Education (NRF-2016R1D1A1B01014260).

## Conflict of Interest

The authors declare that the research was conducted in the absence of any commercial or financial relationships that could be construed as a potential conflict of interest.

## Publisher's Note

All claims expressed in this article are solely those of the authors and do not necessarily represent those of their affiliated organizations, or those of the publisher, the editors and the reviewers. Any product that may be evaluated in this article, or claim that may be made by its manufacturer, is not guaranteed or endorsed by the publisher.
